# Real-Time CASSCF Ehrenfest (TD-CASSCF) Modeling of Spintronics in a Tetrathiafulvalene-Based Nitronyl Nitroxide (BTBN) Model: The Competition between Charge Transfer and Spin Permutation

**DOI:** 10.1021/acs.jpclett.6c02508

**Published:** 2026-09-02

**Authors:** Mercè Deumal, Jordi Ribas-Ariño, Michael A. Robb

**Affiliations:** † Departament de Ciència de Materials i Química Física & IQTCUB, Facultat de Química, 16724 Universitat de Barcelona, Martí i Franquès 1, Barcelona E-08820, Spain; ‡ Department of Chemistry, Molecular Sciences Research Hub, 4615 Imperial College London, White City Campus, 80 Wood Lane, W12 0BZ London, United Kingdom

## Abstract

Using real-time all-electron TD-CASSCF/Ehrenfest simulations, we investigate the molecular origin of magnetoresistance in the organic material BTBN, a tetrathiafulvalene-nitronyl nitroxide diradical. The calculations reveal that charge transport and spin dynamics are strongly coupled. In the high-spin state, where localized spins are aligned in parallel, charge propagates efficiently through the π-stacked system via coherent wave-like migration. In contrast, in low-spin states containing antiparallel spin alignments, the charge migration competes with the spin-permutation processes between localized radical spins. These additional spin-exchange pathways do not transfer charge but divert population away from the charge transport channel, reducing charge-transfer efficiency by roughly 50%, in the example studied, relative to the high-spin state. The simulations therefore identify the competition between electron migration and spin permutation as the fundamental molecular mechanism responsible for magnetoresistance in BTBN, explaining why magnetic-field-induced spin alignment enhances charge transport and lowers electrical resistance.

## Graphical abstract

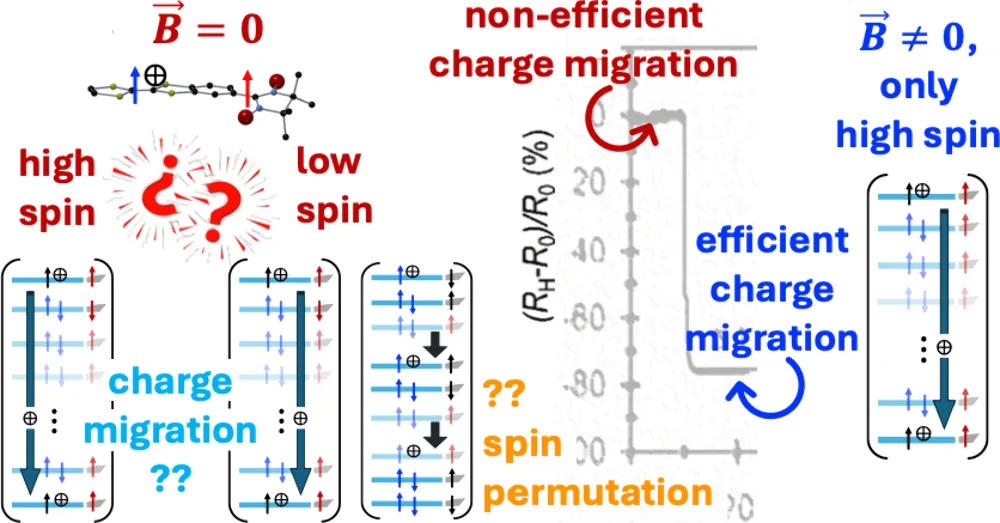

Magnetoresistance (MR) is defined as the change of electrical resistance of a material arising from an externally applied magnetic field and is a central phenomenon in spintronics.^1^ In this paper, we demonstrate a specific molecular mechanism underlying this effect. In particular, in a system displaying some antiparallel spin-alignment, the spin-recoupling (permutation) processes compete with charge propagation, which can hinder efficient charge transfer. In contrast, when a magnetic field drives the system into a parallel spin-aligned state, where spin recoupling cannot take place, charge transfer occurs exclusively.

The MR phenomenon is poised to play a pivotal role in the development of next-generation spintronic devices.^2–4^ Fully harnessing its potential, however, requires a detailed and unbiased understanding of the underlying mechanisms. Many of the existing models impose predefined assumptions about the underlying physics, thereby constraining the actual description of the MR phenomena.^5–7^ Here, we transcend this level by employing real-time all-electron dynamics, a framework that imposes no *a priori* constraints and introduces no free parameters, thereby providing direct access to genuine mechanisms.

The conventional concepts involved in rationalizing the behavior of MR in molecular materials^8–14^ involves exchange and spin delocalization processes,^8^ which in turn rely on the coexistence of units hosting mobile charge carriers and localized paramagnetic spins. Within this framework, BTBN^11^ (see Figure 1), formed by the covalent coupling of tetrathiafulvalene (TTF) and nitronyl nitroxide (NN) units, provides a paradigmatic example of a purely organic material that gives rise to MR upon hole injection through electrodes. It should be noted that concepts such as exchange involve matrix elements of the full electronic Hamiltonian. The usual way to interpret the MR phenomenon in these systems is through the interplay of three physical processes: (1) hopping of mobile electrons between neighboring TTF sites, (2) ferromagnetic (FM) exchange interaction between the localized NN spin and the TTF-based spin carrier within a given BTBN,^11,14^ and (3) weak or zero magnetic couplings between localized NN···NN spins, so that the alignment of each spin is mainly controlled by the external magnetic field. The processes (2) and (3) are realized in a time independent approach, by computing the corresponding matrix elements of the Hamiltonian (for say a dimer of molecules). Here, instead, we will study the MR of BTBN through the lens of real-time all-electron dynamics to provide a qualitative picture of the molecular movements of charge-carriers and dynamics of localized spins that give rise to MR. The Hamiltonian in this time dependent approach contains all the matrix elements of the time independent approach, but we will use several molecular units, and the magnitude of these matrix elements will depend on the detailed molecular structure.

**Figure 1:**
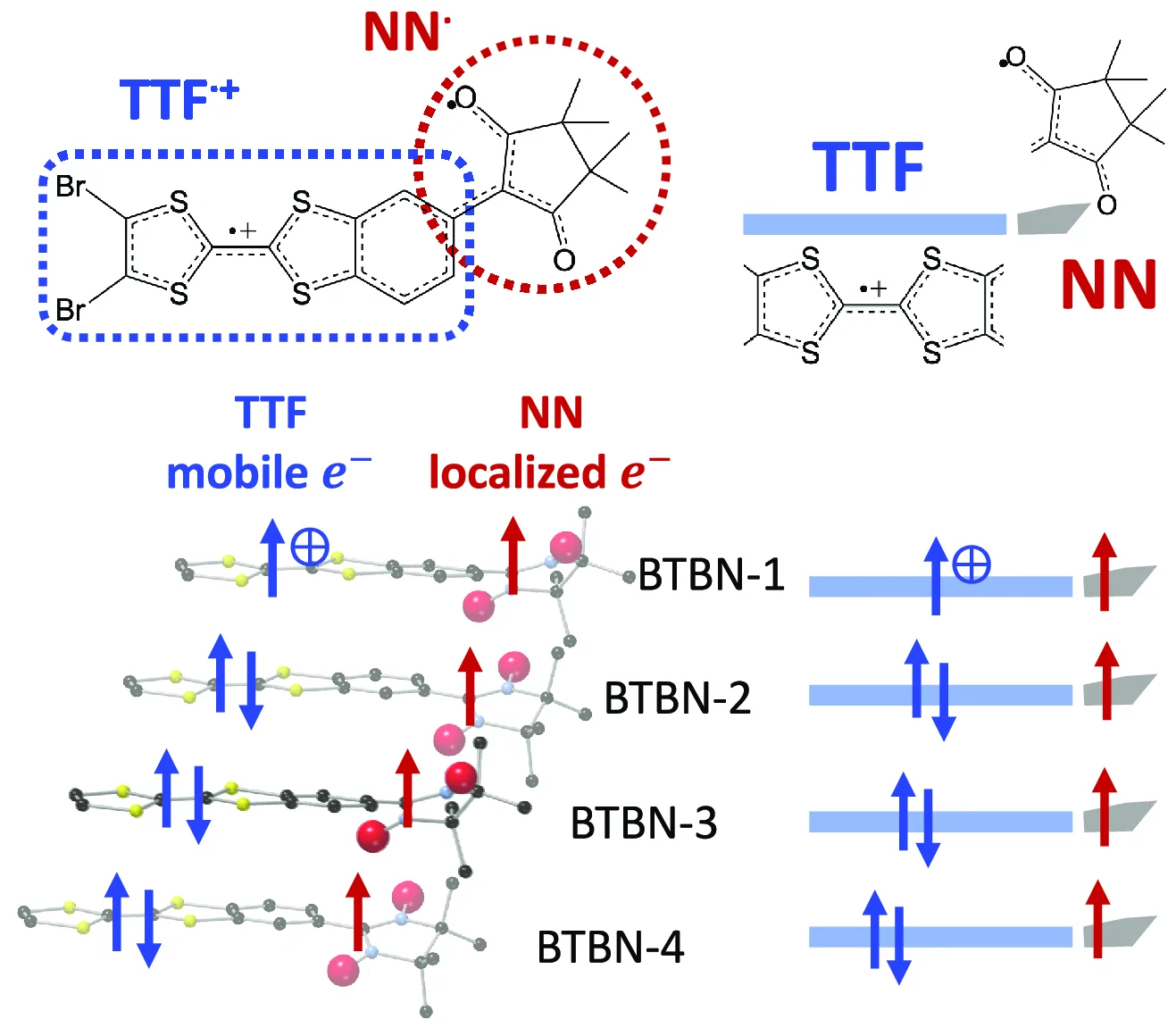
Chemical structure of BTBN diradical unit, consisting of a TTF unit bonded to an NN moiety (top), and π-stack of four BTBNs together with corresponding schematic representation illustrating the arrangement of TTF mobile and NN localized electrons in a high-spin configuration (bottom).

We now begin the description of a rather different mechanism demonstrated in this paper. We shall illustrate it with a π-stack of BTBN units containing a single hole initially localized on the upper unit, with all unpaired electrons aligned parallel, i.e., in a high-spin (sextet) configuration (see Figure 2, where mobile electrons are indicated in black). The electron dynamics will be simulated by solving the time-dependent Schrödinger equation (TDSE see eq 1). In such an approach, one would expect the hole to migrate from the upper to the lower unit along the π-stack without spin flipping (see the mobile electron in black in Configuration State Functions CSF #1 to #3 in Figure 2a, which are chosen to be spin-eigenfunctions; note #1–3 denote the BTBN units in which the hole is localized). Now consider the same π-stack with one NN spin flipped, resulting in a low-spin (quartet) configuration. In this case, two distinct types of dynamics could be expected upon solving the TDSE. First, the hole may migrate from the upper to the lower unit, as in the high-spin case (see the mobile electron in black in CSF #1 to #3 in Figure 2b). Second, a spin permutation may occur without charge transfer, with the hole remaining localized on upper BTBN-1 and the localized NN spins undergoing inversion (see black electrons in Figure 2c). Such a spin permutation may also occur between nonadjacent spins (see Figure 2d). These spin permutations hinder charge migration by introducing competing pathways to the primary charge-transfer process, thereby reducing electron transport compared to the spin-aligned high-spin state. Such distinct electron dynamics are here proposed to constitute the molecular origin of MR in BTBN, since the application of a magnetic field stabilizes the high-spin state and, thus, enhances hole migration.

**Figure 2:**
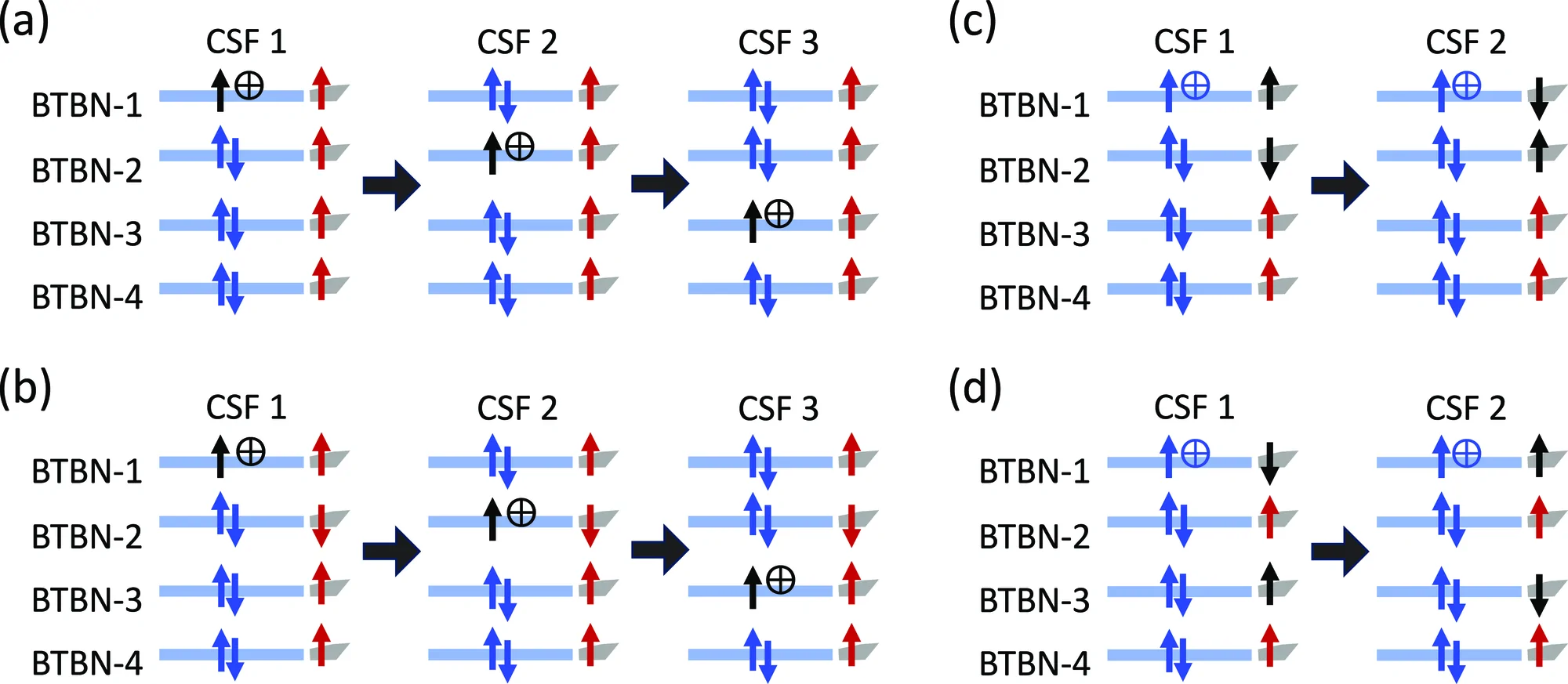
Examples of electron dynamics observed with our TD-CASSCF calculations for charge transfer and spin permutation. In each figure the changing (i.e., mobile) electron configurations are indicated with black electrons and the hole with ⊕. The first column in each configuration (CSF 1, CSF 2, etc.) shows the HOMO-TTF occupations, while the second column in each configuration shows the SOMO-NN occupations (HOMO/SOMO = Highest/Singly Occupied Molecular Orbital). The charge always moves between TTF units and spin permutation takes place between NN units. (a) Sextet state where NN shows a “*αααα*” spin arrangement (α refers to spin up and β to spin down). Comparing CSFs 1, 2 and 3, we see that the charge migrates between adjacent TTF units. (b) As for (a) but with a quartet spin state, where NN has a fixed “*αβαα*” spin arrangement through CSFs 1–3 illustrating charge transfer with hole moving from BTBN-1 to −3 with no spin permutation. (c) Spin permutation of spin array from *ααβα* to *αααβ* in NN, which competes with (b). (d) Spin permutation between nonadjacent NN localized electrons, namely, *αααβ* to *αβαα*.

We now turn to the discussion of electron and spin dynamics of BTBN which has been investigated using real-time dependent CASSCF within the Ehrenfest framework^15^ on a model system consisting of four fragment units excised from the experimental crystal structure.^11^ The crystal of BTBN is made of neutral molecules, i.e., there are no counterions. The TTF radical cation is generated when holes are injected to the material from the electrodes. A snapshot is given in the Supporting Information. As demonstrated previously,^16^ this method and system size provide an accurate description of charge migration. For computational efficiency, each BTBN unit has been simplified by replacing the Br atoms and aliphatic side chains with F and H atoms, respectively. Nuclear positions have been frozen during the 10 fs electron dynamics simulations, as structural changes on this ultrafast time scale are known to be negligible.^16^

In the real-time CASSCF,^15^ (implemented in a development version of Gaussian^17^), rather than use the adiabatic states of the diagonalized Hamiltonian, we introduce a set of coefficients *C*(*t*_*i*_) that mix the adiabatic states which are then propagated as the iterative solution of the time dependent Schrödinger equation (TDSE), where *Ĥ*(*t*_*i*_) is the CASSCF CI Hamiltonian. Equation 1 is solved using the time evolution operator. Full implementation details and derivation, including gradients and second derivatives, are given in ref 15.

$$iĊ\left(t_{i}\right)=Ĥ\left(t_{i}\right)C\left(t_{i}\right)$$ 1

To solve eq 1, one must give the initial conditions (i.e., the coefficient vector *C*(*t*_0_)). This, in turn, corresponds to choosing an initial configuration for the four BTBN unit model system (CSF 1 in Figure 2). Bearing in mind that BTBN is made of one TTF and one NN moieties, and that the charge migration takes place via hole hopping between π-stacked TTF units, one then proceeds as follows. The initial configuration will be the cation corresponding to CSF 1 where the subsequent electron migration involves the HOMO of the TTF unit, and the spin permutation involves the SOMO of the NN radical (see Figure 2 for examples).

We use a CASSCF active space of 8 orbitals (4 TTF HOMOs and 4 NN SOMOs) and 11 electrons (three BTBN^•^ radicals holding 3 electrons each and one BTBN^+••^ diradical holding 2 electrons, resulting in 5 unpaired electrons). A sextet state represents the state in which all spins are aligned parallel (high spin), whereas a quartet state is used to represent a state in which 2 spins align antiparallel. The electron and spin dynamics has been monitored using the computed weights of the configuration state functions (CSF) built from localized molecular orbitals of the time dependent wave functions (eq 1) as a function of time at CASSCF­(11,8)/6–31G* level.^17,18^ The algorithm for implementation of eq 1 using the time evolution operator is fully described using matrix algebra in the Supporting Information of ref 19 and the original formulation of Park and Light.^20^ The orbital localization was carried out using the Boys algorithm.^21^ Thus, the electronic couplings between the localized molecular orbitals in the CSF are the matrix elements of *Ĥ*(*t*_*i*_) (eq 1), i.e., the CASSCF CI Hamiltonian, computed as described in ref 22.

The dynamics (eq 1) initialized on the sextet state (corresponding to Figure 2a) starts from an initial configuration in which the positive charge is located on the TTF of top BTBN-1 unit (CSF 1). Propagating from this initial state, the computed population is transferred from top BTBN-1 (CSF 1) to bottom BTBN-4 (CSF 4) in a wavelike fashion, as shown in Figure 3a. A similar behavior is observed when using an equivalent initial configuration within a quartet state (Figure 2b), where the antiparallel aligned electrons belong to adjacent NN units in BTBN-1 and BTBN-2 and the TTF-mobile electron to the top BTBN-1 unit (CSF 1). The resulting electron dynamics is shown in Figure 3b. In this case, BTBN-1 hosts two parallel electrons which become antiparallel in BTBN-2 upon β-electron transfer to BTBN-1 with the overall spin remaining a quartet. However, although the charge migration is still of a wavelike type, after 4.5 fs, the population of the top BTBN-1 (CSF 1) has decayed by ∼50% less, in comparison to the sextet state. This can be viewed as the electron dynamics manifestation of the magnetoresistance phenomenon. The reason for the population reduction turns out to be simple: the quartet state can experience spin permutation which competes with electron transfer as shown in Figure 4. In the simulation, the spin permutation is observed to take place between localized electrons on NN1 and NN3 because of the slippage of the molecular BTBN units, as previously observed in the case of bisdithiazolyl radicals.^16^ Here, the NN moieties are aligned with a large electronic Hamiltonian matrix element *K*_*ij*_ (see eq 2).

**Figure 3:**
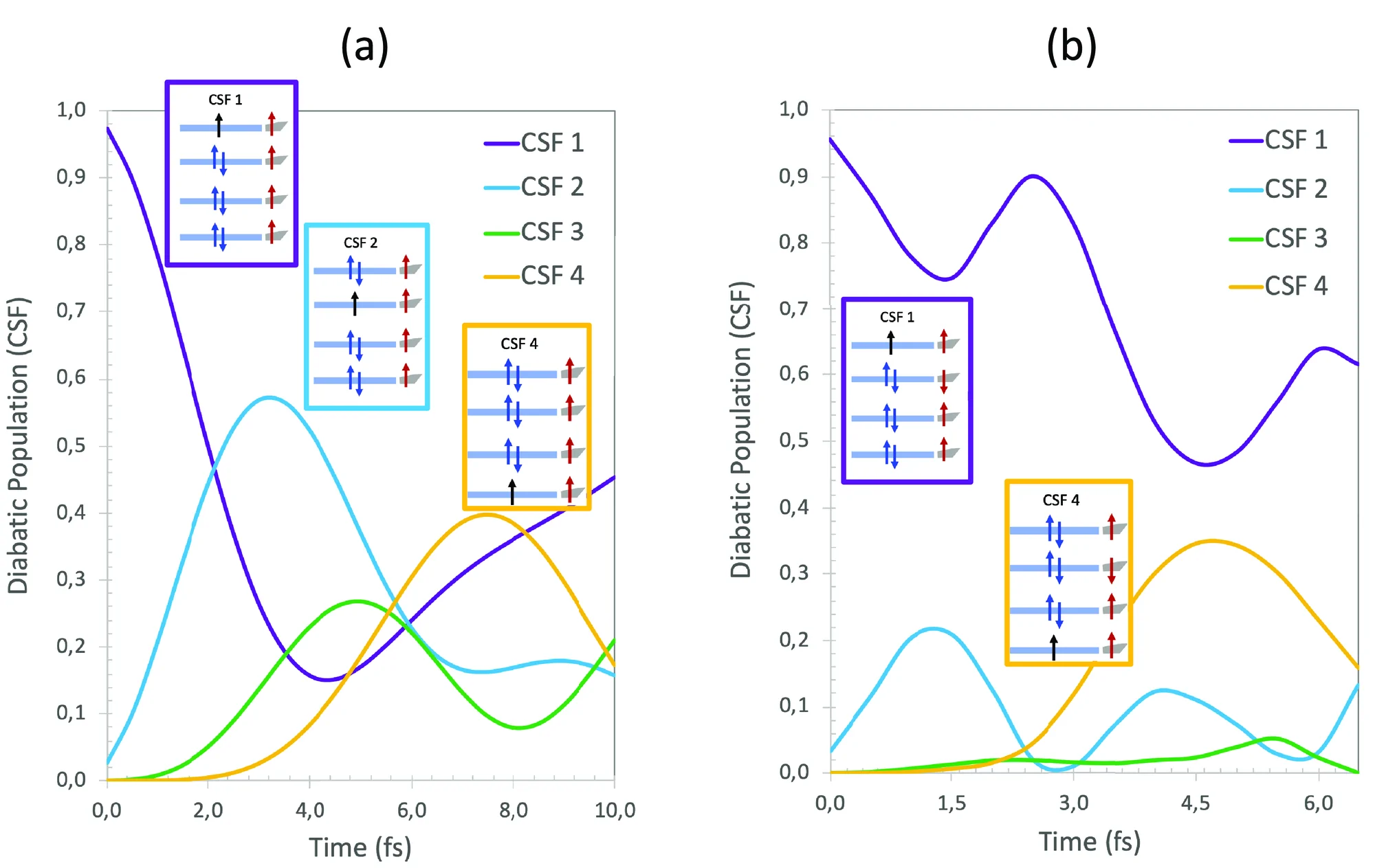
Time evolution of the populations of the diabatic states (configuration state functions, CSF) associated with the (a) sextet and (b) quartet states of the BTBN tetramer model. In each diabatic state, the positive charge is localized on a specific BTBN-unit, namely, BTBN-1 in CSF 1 (purple), BTBN-2 in CSF 2 (blue), BTBN-3 in CSF 3 (green), and BTBN-4 in CSF 4 (orange). Note that the population reached by CSF 2 through CSF 4 is different for sextet and quartet states.

**Figure 4:**
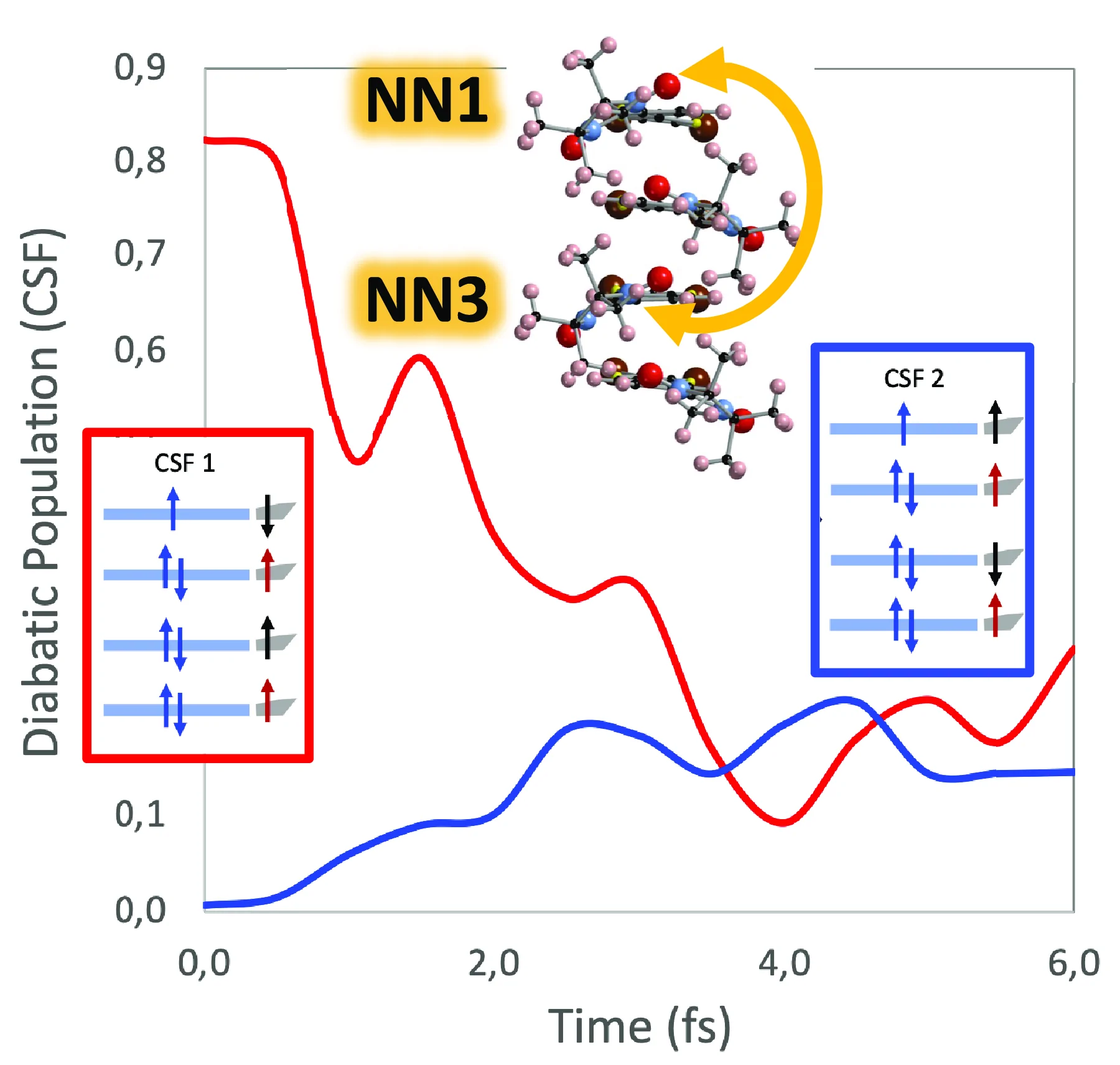
Time evolution of the populations of two diabatic states associated with the quartet spin state of the BTBN tetramer model with positive charge localized on BTBN-1 to illustrate the spin permutation taking place between NN1 and NN3. Other possible spin permutations among NN1–4 are an order of magnitude smaller.

The possible charge transfer and spin permutations that can occur in BTBN, namely, electron migration between TTF units within the sextet and quartet states (Figure 3), and spin permutation between NN units within quartet states (Figure 4), are controlled by the electronic Hamiltonian matrix elements shown in eq 2 (where orbitals *i* and *j* are the orbitals that differ between CSF 1 and CSF 2)

2 $$K_{ij}=\langle i\left|j\right\rangle +\langle i\mathrm{j̅}|\left|i\mathrm{j̅}\right\rangle -\langle i\mathrm{j̅}|\left|j\mathrm{i̅}\right\rangle$$

The first term in eq 2 is the usual one-electron orbital coupling between orbitals *i* and *j*, the second terms is the Coulomb integral for the pair *i* *j̅* and the final term is the exchange integral between *i* *j̅* and *j* *i̅*.

In Figure 2a, all three terms contribute; however, for the spin permutation in Figure 2d, only the third two-electron-exchange term contributes. It is worth mentioning that the spin permutation in Figures 2c and 2d does not yield an electron/charge transfer. Thus, this process is in competition with the charge transfer. One cannot *a priori* have both processes because the matrix element would involve more than two spin–orbital differences. Thus, the magnitude of the charge transfer is controlled by the electronic Hamiltonian matrix element *K*_*ij*_ (eq 2), which is dominated by the one-electron term. The spin permutation is controlled by the two-electron-exchange term (last term in eq 2), which in turn is controlled by the differential overlap or interpenetration of molecular orbitals *i* and *j*. The sextet is the classic simple reference case.

Overall, our simulations demonstrate that the MR in BTBN originates in the competition between electron migration and spin permutation (see Figure 5). Specifically, when the localized spins on NN units are parallelly aligned, the mobile electron can be transferred between adjacent TTF units without requiring any spin flip provided its spin aligns antiparallel to those of NNs (see Figure 5a). In contrast, when any two localized spins on the NN units align antiparallel, there is a competition between the migration of the TTF mobile electron and the permutation between two adjacent localized NN spins (Figure 5b). These spin permutations lead to an extra resistance to charge migration, which is detrimental to electron transport and rules MR at the molecular level.

**Figure 5:**
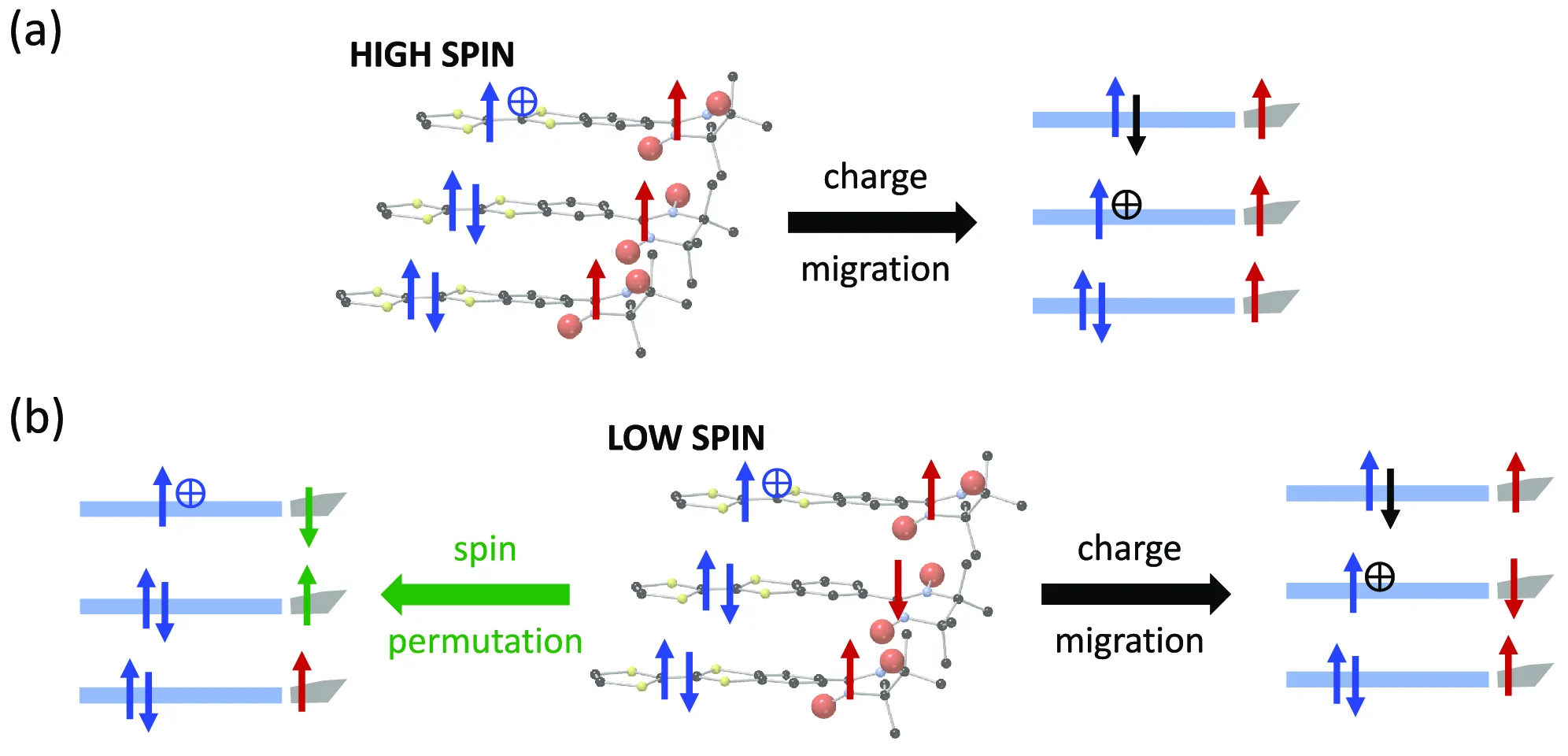
(a) Upon hole injection into the upper BTBN unit, a high spin configuration of three BTBN units can undergo charge migration, whereas (b) a low spin configuration of three BTBN units can undergo either spin permutation or charge migration.

## Supplementary Material



## Notes

Crystal structure of BTBN showing the unit cell containing 8 molecules (Z = 8) (PDF)
