# Supplementary material for: Real-Time CASSCF Ehrenfest (TD-CASSCF) Modeling of Spintronics in a Tetrathiafulvalene-Based Nitronyl Nitroxide (BTBN) Model: The Competition between Charge Transfer and Spin Permutation
Source: J Phys Chem Lett. 2026 Sep 2;17(37):10635–9. doi: 10.1021/acs.jpclett.6c02508 (PMC13592076; doi:10.1021/acs.jpclett.6c02508)

Supplementary Information for

Real-Time CASSCF Ehrenfest (TD-CASSCF) Modelling of Spintronics in a *Tetrathiafulvalene-Based Nitronyl Nitroxide (BTBN) model: The Competition Between Charge Transfer and Spin Permutation*

*Mercè Deumal[1], Jordi Ribas-Ariño[1] and Michael A. Robb[2]**

[1] Departament de Ciència de Materials i Química Física & IQTCUB, Facultat de Química, Universitat de Barcelona, Martí i Franquès 1, Barcelona, E-08820

merce.deumal@ub.edu (orcid Id 0000-0002-6385-8149), j.ribas@ub.edu (orcid Id 0000-0003-4088-6187)

[2] Department of Chemistry, Molecular Sciences Research Hub, Imperial College London, White City Campus, 80 Wood Lane, W12 0BZ, London, United-Kingdom

Mike.Robb@imperial.ac.uk (orcid Id 0000-0003-0478-8301)

Crystal structure of BTBN showing the unit cell containing 8 molecules (Z=8). The BTBN molecules are arranged in stacks along the *c*-axis. The red arrow highlights one such π-stack, which is shown alongside the unit cell to illustrate the molecular arrangement within a stack of four BTBN units.

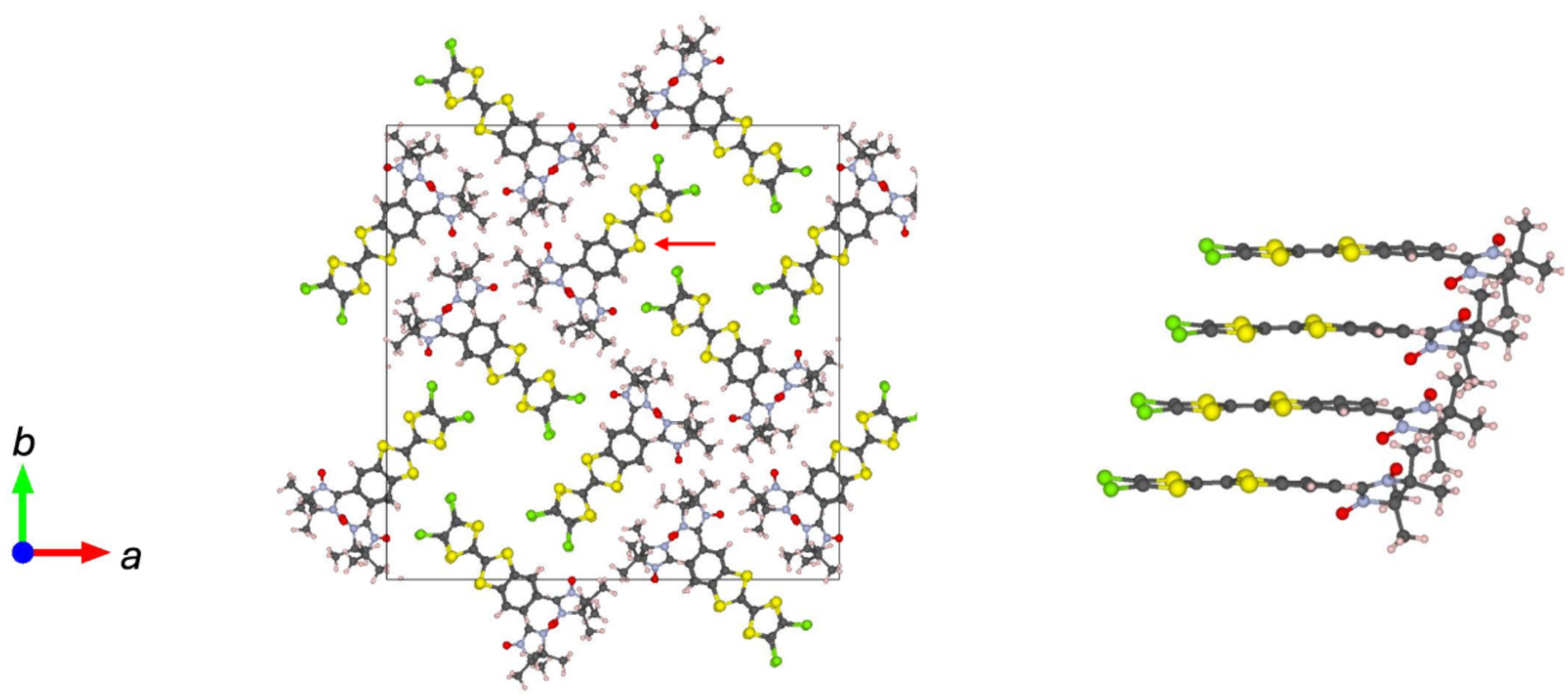

Supplement: Supplementary file 1 [file jz6c02508_si_001.pdf]
